# Improvement in breast cancer survival across molecular subtypes in Hungary between 2011 and 2020: a nationwide, retrospective study

**DOI:** 10.3389/fonc.2025.1465511

**Published:** 2025-03-31

**Authors:** Miklós Darida, Gábor Rubovszky, Zoltán Kiss, Borbála Székely, Balázs Madaras, Zsolt Horváth, Judit Kocsis, István Sipőcz, Máté Várnai, Éva Balogh, Krisztina Kovács, Viktória Buga, Eugenia Karamousouli, Tamás G. Szabó, György Rokszin, Ibolya Fábián, István Kenessey, András Wéber, Péter Nagy, Zsófia Barcza, Krisztina Bogos, Zoltán Vokó, Csaba Polgár

**Affiliations:** ^1^ MSD Hungary, Budapest, Hungary; ^2^ Department of Oncological Internal Medicine and Clinical Pharmacology "B" and National Tumor Biology Laboratory, National Institute of Oncology, Budapest, Hungary; ^3^ Second Department of Medicine and Nephrology-Diabetes Centre, University of Pécs Medical School, Pécs, Hungary; ^4^ Department of Oncology, Bács-Kiskun County Teaching Hospital, Kecskemét, Hungary; ^5^ Aladár Petz County University Teaching Hospital, Győr, Hungary; ^6^ MSD, Athens, Greece; ^7^ RxTarget Ltd., Szolnok, Hungary; ^8^ University of Veterinary Medicine, Budapest, Hungary; ^9^ Department of Pathology, Forensic and Insurance Medicine, Semmelweis University, Budapest, Hungary; ^10^ Hungarian National Cancer Registry and National Tumor Biology Laboratory, National Institute of Oncology, Budapest, Hungary; ^11^ Department of Molecular Immunology and Toxicology and the National Tumor Biology Laboratory, National Institute of Oncology, Budapest, Hungary; ^12^ Department of Anatomy and Histology, HUN-REN–UVMB Laboratory of Redox Biology Research Group, University of Veterinary Medicine, Budapest, Hungary; ^13^ Chemistry Coordinating Institute, University of Debrecen, Debrecen, Hungary; ^14^ Syntesia Medical Communications Ltd, Budapest, Hungary; ^15^ National Korányi Institute of Pulmonology, Budapest, Hungary; ^16^ Center for Health Technology Assessment, Semmelweis University, Budapest, Hungary; ^17^ Syreon Research Institute, Budapest, Hungary; ^18^ National Institute of Oncology and the National Tumor Biology Laboratory, Budapest, Hungary; ^19^ Department of Oncology, Semmelweis University, Budapest, Hungary

**Keywords:** breast cancer, breast cancer subtypes, survival, nationwide, retrospective, epidemiology

## Abstract

**Background:**

Despite well-documented clinical differences across breast-cancer (BC) molecular subtypes and relevant changes in therapeutic interventions over the past decades, there remains a significant lack of up-to-date epidemiologic data and real-world outcomes, particularly in Central and Eastern Europe.

**Methods:**

This was a nationwide, retrospective study using the claims databases of the Hungarian National Health Insurance Fund (NHIF) that included patients who were newly diagnosed with BC between 2011 and 2020. BC subtypes were defined based on the therapies received. Overall survival (OS) and net survival rates were calculated.

**Results:**

Between 2011 and 2020, 74,143 patients were newly diagnosed with BC based on ICD-10 diagnostic codes in the NHIF database and 80.1% of the cases could be classified into subtypes based on therapy. The most common subtype was HER2–/HR+ BC, identified in 61.9% of patients, followed by triple negative breast cancer (TNBC) in 8.4%, HER2+/HR+ BC in 6.2%, and HER2+/HR- BC in 3.6% of cases. The proportions of TNBC and HER2+/HR+ were higher among younger patients, than in elderly cohorts. The 5-year OS of the total BC population was 74.2% in patients diagnosed between 2015–2019. Patients with TNBC had the poorest 5-year OS (TNBC: 61.4%; HER2+/HR+: 86.5%; HER2-/HR+: 79.1%; HER2+/HR–: 71.9%). Net survival rates (i.e. survival rates after adjusting the effects of other causes of death) varied across diagnostic periods and molecular subtypes. In most cases, patients diagnosed later during the study period tended to have numerically better survival rates. Patients with HER2–/HR+ BC had the most favorable net survival, with 5-year net survival exceeding 92% during the whole observation period, while TNBC patients had the lowest 5-year net survival rates ranging between 63.6% and 65.8% during the study period.

**Conclusion:**

Our nationwide study describes the distribution and survival of BC patients with different subtypes based on a retrospective analysis of the health insurance fund database. There remains a significant room for improvement in the survival of more aggressive molecular subtypes including HR–/HER2+ and triple-negative BC, which are more common in younger age cohorts.

## Introduction

Breast cancer (BC) is by far the most common cancer type among women worldwide, which accounts for about 2.3 million new cases and 666.000 deaths in 2022 (1) making it the primary contributor to female cancer-related mortality (2). The prognosis of BC has significantly improved over the past few decades, mainly due to the introduction and improvements in chemo-, endocrine and biological therapies in both early and metastatic settings as well as the implementation of effective screening programs allowing for early diagnosis (3). Based on the global CONCORD surveillance program for trends in cancer survival, 5-year net survival rates of BC among women diagnosed between 2010–2014 were ≥80% in the majority of European countries, with the highest survival observed in Northern Europe (4). As a result of improving survival and high incidence, BC is the most prevalent malignancy in Europe (5).

While classification of BC is constantly evolving with scientific advances on the field, its classical subdivision into four distinct clinical subgroups is based on the expression of hormone receptors (HR) and human epidermal growth factor receptor 2 (HER2) in routine clinical practice. From clinical perspective and treatment planning it is essential to distinguish HR+ from HR- and HER2+ from HER2- tumors. Four main groups can be defined according to receptor status: HER2-/HR+, HER2+/HR+, HER2+/HR- and HER2-/HR- (referred to as TNBC) groups (6). The different molecular subtypes of BC have distinct biological features including variability in recurrence and tendency to form metastases (7–9), as well as response patterns to treatments, which has been shown to influence survival (10). TNBC, the subtype with the poorest early prognosis and limited therapeutic options (11), accounts for about 10% of all BC cases. A Surveillance, Epidemiology, and End Results (SEER) database analysis as well as a real-world study from Belgium reported the highest survival rates among women with the HER2–/HR+ subtype, while the worst survival was observed in women with TNBC in both studies (12, 13).

Differences in biological behavior and prognosis across clinical subtypes, as well as the availability of distinct therapeutic options (14, 15) underline the importance of understanding and closely monitoring the epidemiology of major disease subtypes. The Hungarian HUN-CANCER EPI - Multiple Cancer Epidemiology study was launched to assess the incidence and mortality of the 20 most common cancer types in Hungary from 2011, with future extension until 2025. Initial results from this program related to trends in BC incidence and mortality have been already published elsewhere (16). However, the scarcity of data on the epidemiology of distinct BC subtypes and their survival highlights the need for further investigations, especially in Central Eastern Europe, where to the best of our knowledge such comprehensive, nationwide analyzes have not yet been performed. With regards to Hungary specifically, the National Cancer Registry reported overall BC incidence, mortality, and survival data by stages from the period 2001-2015 (17) besides the aforementioned BC epidemiology data from our program. We are aware of a single paper on subtype-level data; however, it reported retrospective data from 490 patients who underwent BC surgery between 2000 and 2007 at one university clinic (18).

Therefore, the current nationwide, retrospective analysis of the HUN- CANCER EPI study was performed to assess the age-related incidence of BC subtypes in Hungary and explore differences in short- and long-term survival of these molecular subtypes in comparison with available international sources. Furthermore, we aimed to explore the differential survival outcomes among TNBC patients based on their initial treatment approach, given that TNBC is recognized as the subtype with the poorest prognosis.

## Materials and methods

### Data sources

Data are collected as part of the HUN- CANCER EPI nationwide, retrospective study which was conducted using the database of the Hungarian National Health Insurance Fund (NHIF) to characterize the current epidemiology of malignant diseases in Hungary. The NHIF is a comprehensive database of ICD-10 codes related to reimbursed prescription claims, in- and outpatient visits and medical procedures, covering virtually the whole population as there is no other insurance system covering BC care in Hungary. Thus, leveraging the NHIF database allows for comprehensive analyses pertaining to BC care.

Our analysis on breast cancer included female patients diagnosed with BC between January 1, 2011 and December 31, 2020. Case definitions were based on occurrences of ICD-10 diagnostic codes entered in the NHIF database. screening period was set from 2009 to 2010 to accurately identify newly diagnosed BC patients and exclude those with prevalent BC who had a prior diagnosis of BC at the start of the study period. BC patients were primarily defined as patients who had at least two occurrences of BC-related ICD-10 codes, i.e. codes under C50 (of note, *in situ* carcinoma cases are coded elsewhere). Furthermore, patients who died within 60 days after the date of the first C50 ICD-10 diagnosis code were also included in the analysis, even if the code occurred only once. Certain cases contained claims data with multiple ICD-10 cancer-related codes accumulating over time until September 30, 2022, set as the data collection cut-off date. Within the framework of the HUN- CANCER EPI study, cancer types were identified and assigned to patients based on the predominantly occurring cancer-related ICD-10 codes, hence, a patient with different cancer related ICD-10 codes was identified as breast cancer patient, if C50 code was recorded in the majority of ICD-10 code entries (still, 82% of BC cases analyzed had only C50 ICD-10 code without any other type of cancer related codes).

NHIF database records do not directly capture different molecular subtypes, however, specific treatments against HER2, and HR expressing tumors allow for the identification of patients with receptor expressing subtypes and TNBC. Therefore, we classified patients based on therapies received. Patients who were prescribed trastuzumab, trastuzumab emtansine, pertuzumab, or lapatinib with the ICD-10 code C50 as the main diagnosis at least once were classified as having HER2+ BC. Patients with HR+ BC were identified based on at least one prescription for tamoxifen, anastrozole, letrozole, exemestane, fulvestrant, GnRH analogues, abemaciclib, palbociclib, ribociclib, or everolimus with C50 as the main diagnosis code during the same period. We also created a HER2+/HR+ subgroup using both BC treatment types previously mentioned for HER2+ and HR+ subtypes. Patients who did not receive treatment for HER2+ or HR+ BC but received chemotherapy at least once with C50 as the main diagnosis code were regarded as TNBC patients. For TNBC subtype analyses, patients were further classified based on the timing of systemic chemotherapy 60 days within BC surgery. Namely, cases with systemic chemotherapy administered after the first cancer related code, but before the coded BC-related surgical intervention were defined as TNBC patients starting their treatment with neoadjuvant therapy while those receiving systemic chemotherapy following BC-related surgery were classified as adjuvant treatment recipients for survival analyses and the terms “neoadjuvant and “adjuvant”, respectively, will be used to refer to these patient groups throughout this paper. In case of those patients, where neoadjuvant and adjuvant treatment were both identified, persons were grouped in the neoadjuvant sub-group.

### Statistical analysis

Data collected were anonymous and non-identifiable. Number of newly diagnosed BC cases with mean age as well as the distribution of patients according to age and type of BC were determined for the total time period and for the different diagnostic intervals (2011–2012; 2013–2014; 2015–2016; 2017–2018; 2019; 2020). As the COVID-19 epidemic emerged in Europe in 2020, we reported data for 2019 and 2020 separately.

To describe clinical outcomes, overall survival was determined for each breast cancer subtype. Overall survival rates were presented according to the Kaplan-Meier method for each half of the observation period separately and changes were assessed by Cox proportional hazards regression analysis. Given its poor prognosis among subtypes, we also evaluated overall survival data on TNBC specifically by treatment strategies, i.e. whether it was started in the neoadjuvant or adjuvant setting. In addition, overall survival for subtypes were also calculated for 2-year diagnostic cohorts in each age group.

While overall survival carries key prognostic information, it reflects survival related to both cancer and any other possible cause of death. To evaluate the changes in the effect of having breast cancer on survival and to enable international comparisons, one to five-year net survival rates were estimated to enable international comparisons. Net survival corresponds to the cumulative probability of surviving up to a given time since diagnosis (e.g., 5 years) after correcting for other causes of death (as background mortality). Net survival was calculated using the unbiased, non-parametric Pohar Perme estimator which is the gold standard for estimating net survival (19). Briefly, the Pohar Perme method utilizes life table data to provide an estimate of survival probabilities directly attributable to the disease. The method determines the excess mortality rate for individual patients explicitly considering both the observed survival of patients and the expected survival calculated from population data. Background mortality data for the calculation were derived from life tables published by the Hungarian Central Statistical Office (accessed at http://www.lifetable.de). Patients over the age of 100 years were excluded from the net survival analysis, cases reaching it were censored at 100 years of age.

All calculations were performed using R version 3.6.1 (05/07/2019) with package boot version 1.3-20. Figures were generated in Microsoft Excel. Ethical permission was provided by the Medical Research Council (IV/298-2/2022/EKU).

## Results

Between 2011 and 2020, altogether 74,143 patients were newly diagnosed with BC based on the NHIF database. Population characteristics by diagnostic periods are presented in **Table 1**. The number of newly diagnosed BC patients remained in a similar range during the study period, however, the number of patients was lower in 2020, than in 2019 (6,452 versus 7,305 -11.7%). The mean age was 63.13 years at the time of diagnosis, without apparent trends throughout the observation period. In general, the number of new cases increased with age: 18.7% of patients were aged <50 years, 18.9% were 50–59 years old, 28.2% were 60–69 years old, and 34.2% were older than 70 years. Of note, the proportion of patients younger than 50 years increased, which was accompanied by a decrease in the proportion of patients aged 50–59 years. The proportion of patients above 70 years of age also increased slightly. Number of patients by age group and BC subtype is provided in **Supplementary Table 1**. In addition to the BC cases in females described above, 799 male BC patients were also observed during the study period (range of annual cases: 71-96), these cases have not been included in the analyses.

**Table 1:** Number of newly diagnosed BC patients in Hungary between 2011 and 2020 according to age and subtype of BC.

	Characteristics of Patients													
	2011-2012		2013-2014		2015-2016		2017-2018		2019		2020		2011-2020	
Patients with new BC diagnosis (n)	15,138		15,012		15,185		15,051		7,305		6,452		74,143	
Mean age at diagnosis (y, mean ± SD)	63.32 ± 13.74		63.42 ± 13.6		63.12 ± 13.9		62.95 ± 14.04		63.08 ± 14.02		62.51 ± 14.1		63.13 ± 13.87	
Age groups	n	%	n	%	n	%	n	%	n	%	n	%	n	%
0-39	751	5.0%	727	4.8%	760	5.0%	814	5.4%	356	4.9%	323	5.0%	3,731	5.0%
40-49	1,763	11.6%	1,820	12.1%	2,123	14.0%	2,197	14.6%	1,097	15.0%	1,110	17.2%	10,110	13.6%
50-59	3,284	21.7%	3,041	20.3%	2,701	17.8%	2,556	17.0%	1,308	17.9%	1,094	17.0%	13,984	18.9%
60-69	4,251	28.1%	4,274	28.5%	4,396	28.9%	4,326	28.7%	1,969	27.0%	1,711	26.5%	20,927	28.2%
70<=	5,089	33.6%	5,150	34.3%	5,205	34.3%	5,158	34.3%	2,575	35.2%	2,214	34.3%	25,391	34.2%
Type of BC	n	%	n	%	n	%	n	%	n	%	n	%	n	%
HER2+/HR-	529	3.5%	485	3.2%	537	3.5%	559	3.7%	282	3.9%	267	4.1%	2,659	3.6%
HER2-/HR+	8,935	59.0%	9,188	61.2%	9,500	62.6%	9,434	62.7%	4,680	64.1%	4,122	63.9%	45,859	61.9%
HER2-/HR- (TNBC)	1,342	8.9%	1,329	8.9%	1,236	8.1%	1,226	8.1%	543	7.4%	565	8.8%	6,241	8.4%
HER2+/HR+	796	5.3%	809	5.4%	996	6.6%	1,032	6.9%	523	7.2%	451	7.0%	4,607	6.2%
BC - no SACT data	3,536	23.4%	3,201	21.3%	2,916	19.2%	2,800	18.6%	1,277	17.5%	1,047	16.2%	14,777	19.9%
TNBC	n	%	n	%	n	%	n	%	n	%	n	%	n	%
TNBC Neoadjuvant	271	20.2%	292	22.0%	325	26.3%	428	34.9%	242	44.6%	244	43.2%	1,802	28.9%
TNBC Adjuvant	515	38.4%	500	37.6%	430	34.8%	342	27.9%	122	22.5%	139	24.6%	2,048	32.8%
TNBC Other	556	41.4%	537	40.4%	481	38.9%	456	37.2%	179	33.0%	182	32.2%	2,391	38.3%

BC, breast cancer; HER2, human epidermal growth factor receptor 2; HR, hormone receptor; SACT, systemic anticancer therapy; SD, standard deviation; TNBC, triple-negative breast cancer, TNBC neoadjuvant: received systemic therapy within 60 days before surgery, TNBC adjuvant: received systemic therapy within 60 days after surgery, TNBC Other: received systemic therapy and not falling in the adjuvant or neoadjuvant categories.

The most common subtype of BC was HER2–/HR+, identified in 61.9% of patients, while TNBC accounted for 8.4% of all BC cases. HER2+/HR+ and HER2+/HR– BC represented 6.2% and 3.6% of cases, respectively. About 19.9% of patients with BC in the whole study period could have not been classified due to the lack of systemic anticancer therapy (SACT) data. Leaving out these patients and calculating proportions only for those records that allowed subtype classification, HER2-/HR+ patients represented 77.2%, the proportion of the HER2+/HR+ group was 7.8%, HER2+/HR- was 4.5%, while 10.5% fell in the TNBC group.

**Figure 1:**
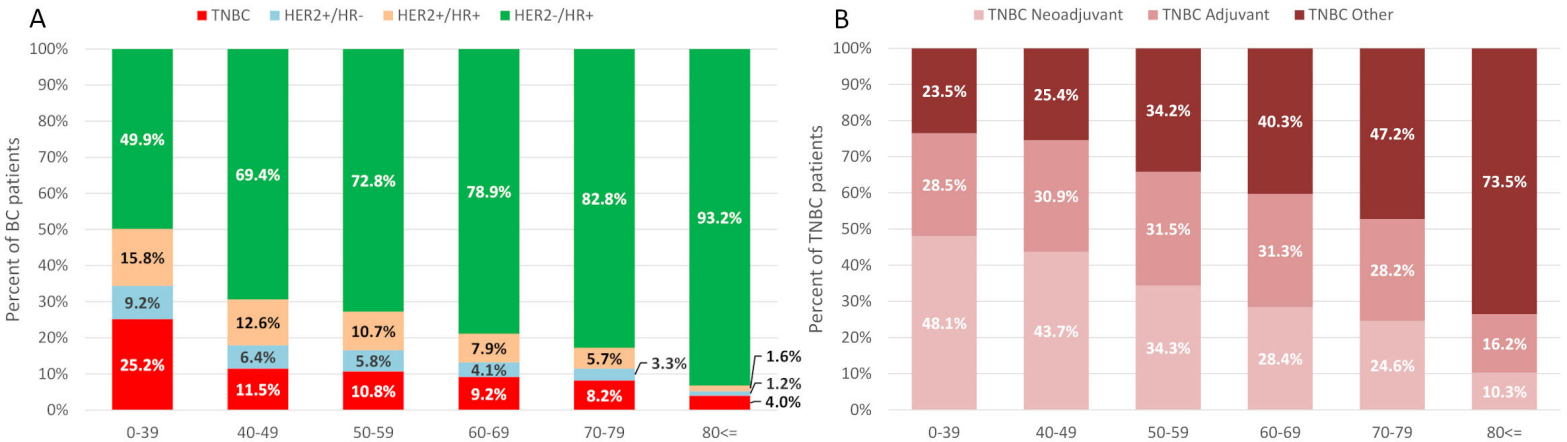
Proportion of BC subtypes by age group **(A)** and proportion of TNBC patients receiving systemic therapy in different settings (neoadjuvant, adjuvant, other systemic therapy not falling in the previous 2 categories) **(B)** in the 2015-2019 diagnostic cohorts. BC, breast cancer; HER2, human epidermal growth factor receptor 2; HR, hormone receptor; TNBC, triple-negative breast cancer.

The proportion of patients with TNBC, HER2+/HR+, and HER2+/HR– malignancies were generally higher in younger age cohorts, with the highest proportion found in patients aged 0–39 years (25.2%, 15.8%, and 9.2% for the TNBC, HER2+/HR+, and HER2+/HR– groups, respectively). The proportion of HR+ tumors increased with age (**Figure 1A**).

Among all patients with TNBC during our study period, 28.9% received initial neoadjuvant therapy (with or without later adjuvant treatment) and 32.8% received adjuvant therapy. Of note, this was not consistent throughout the years: the proportion of neoadjuvant treatment increased from 20.2% in 2011–2012 to 44.6% in 2019. The proportion of adjuvant systemic therapy initiation decreased from 38.4% in 2011–2012 to 22.5% in 2019. Proportion of patients receiving perioperative (pre- and post-surgical) systemic therapy was 58.6%, 59.1%, 59.6%, and 61.1% for the periods 2011-2012, 2013-2014, 2015-2016 and 2017-2018, respectively, for years 2019, 2020 and 2021, the observed percentages were 67.0, 67.8 and 64.9%. The use of neoadjuvant and adjuvant chemotherapy was more frequent in younger age groups, than in elderly cohorts in 2015–2019 (**Figure 1B**), but the use of neoadjuvant treatment tended to increase across all age groups. In TNBC patients aged ≥70 and ≥80 years, 47.2% and 73.5% did not receive any perioperative systemic treatment, respectively.

**Figure 2:**
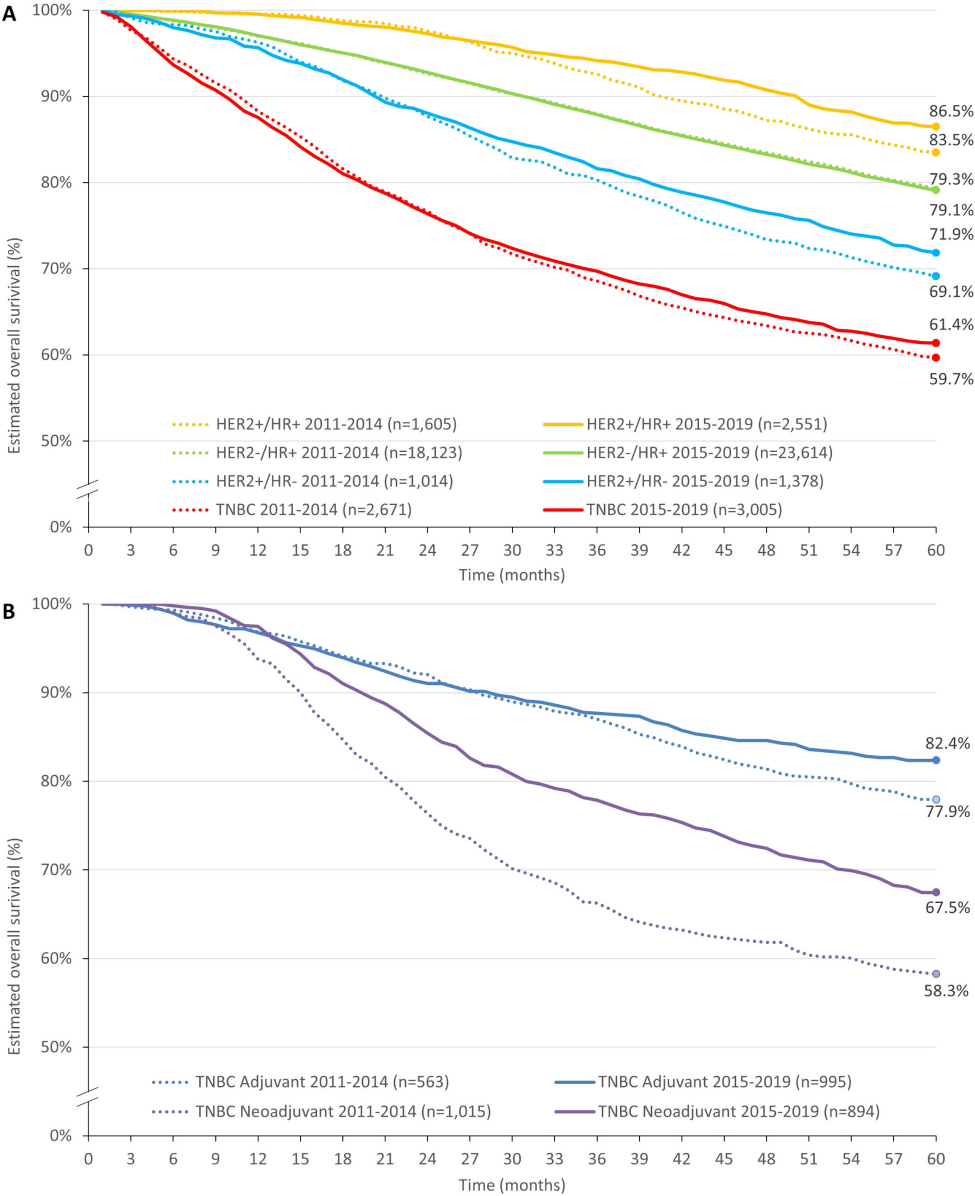
Overall survival of Hungarian BC patients diagnosed between 2015–2019 by cancer subtypes **(A)** and in the TNBC group by treatment strategies (surgery followed by adjuvant systemic treatment; neoadjuvant systemic treatment followed by surgery) **(B)**. BC, breast cancer; HER2, human epidermal growth factor receptor 2; HR, hormone receptor; TNBC, triple-negative breast cancer.

The 5-year overall survival of BC was 73.1% and 74.2% in patients diagnosed in the periods between 2011 and 2014, and 2015 and 2019, respectively. During the whole study period, overall survival was much higher among patients with HR+ BC, than among those with HR– BC. Specifically, in the 2015–2019 diagnostic period, HER2+/HR+ BC had the highest 5-year overall survival (86.5%, 95% CI: 85.0% - 88.0%), while TNBC had the poorest (61.4%, 95% CI: 59.6%-63.2%). Patients with HER2+/HR– BC had a 5-year survival of 71.9% (95% CI: 69.3%-74.4%, **Figure 2A**). These trends were consistent during the 5-year follow-up period. In the TNBC group, the survival rates decreased to 69.74% by year 3 (OS was 87.55% and 76.37% at 12th and 24th months, respectively). Compared with data from patients diagnosed between 2011 and 2014, OS has numerically improved except for the HER2-/HR+ group, in which it remained practically unchanged (79.3% [95% CI: 78.7%-79.9%] 5-year OS in the 2011-2014 and 79.1% [95% CI: 78.5%-79.7%] in the 2015-2019 diagnostic groups), however this was only statistically significant in the HER2+/HR+ subgroup (Hazard ratio: 0.83 (95%CI:0.72–0.96) p= 0.014; HR for HER2-/HR+: 0.99 (95%CI:0.96–1.03; p=0.756), for HER2+/HR-: 0.89 (95%CI:0.77-1.02; p=0.103) and for TNBC: 0.96 (95%CI:0.88–1.04; p=0.284).

**Figure 3:**
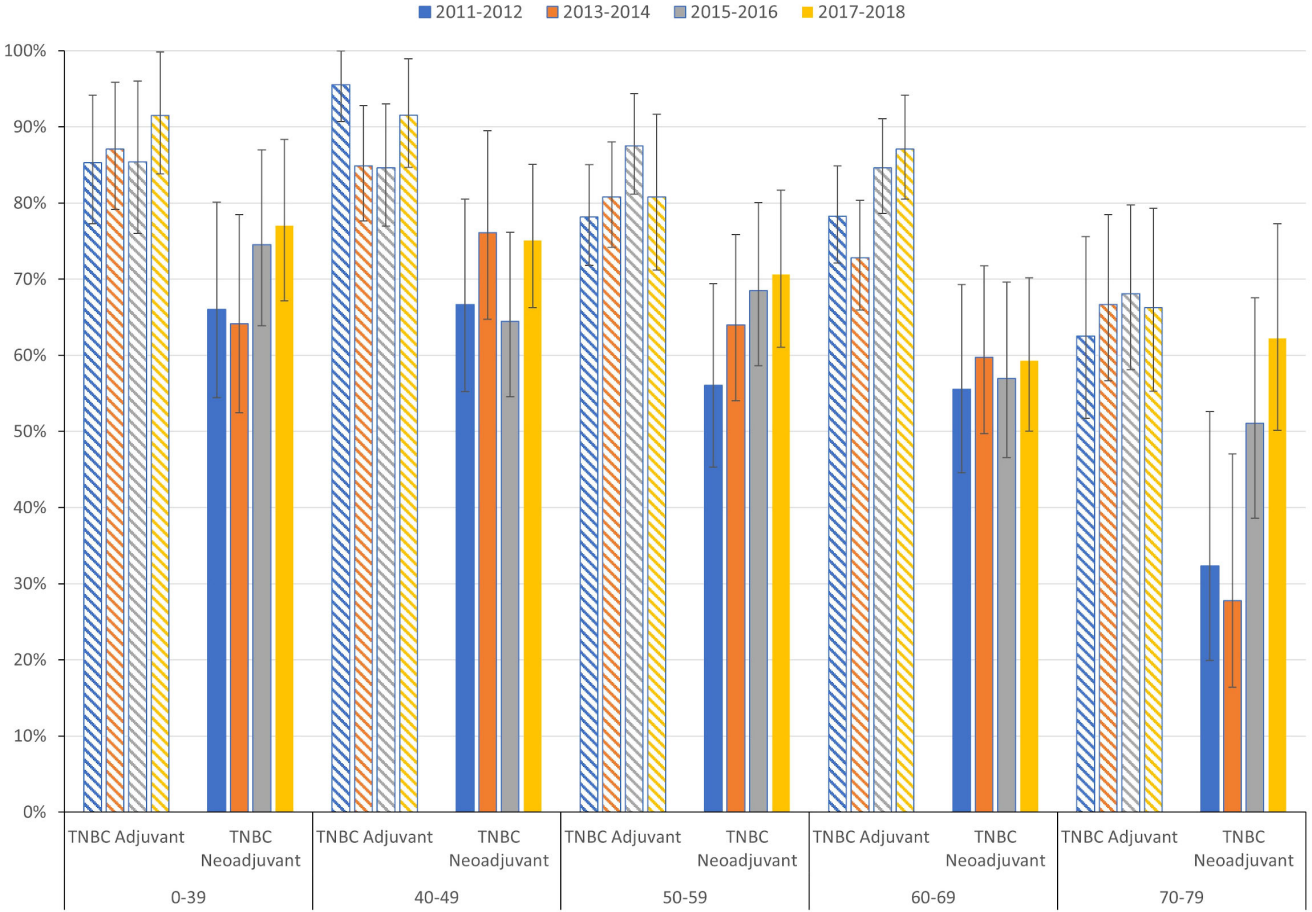
5-year overall survival for patients with triple-negative BC undergoing peri-operative systemic treatment by treatment strategy applied (adjuvant or neoadjuvant) and different diagnostic periods (from 2011–2012 to 2017-2018). Error bars represent 95% confidence intervals.

As seen in **Figure 2B**, in the TNBC group, patients undergoing surgery and receiving adjuvant chemotherapy had remarkably better 5-year overall survival (82.4%, 95% CI: 79.8%-84.9%), than those who received neoadjuvant treatment before surgical intervention (67.5%, 95% CI: 64.3%-70.7%) in the 2015-2019 diagnostic group. Of note, patients receiving neoadjuvant therapy had numerically slightly higher survival rates at 12 months: 97.49% (95% CI: 96.5%-98.5%) for neoadjuvant vs. 96.76% (95% CI: 95.6% -97.9%) for adjuvant therapy at 1-year follow-up (**Supplementary Table 2A**, number of patients at risk are provided in **Supplementary Table 2B**). In comparison to the 2011-2014 diagnostic period, improvement was seen in both treatment settings among those diagnosed between 2015 and 2019, however, this reached statistical significance only in the case of neoadjuvant treatment [adjuvant HR: 0.84 (95% CI:0.69–1.02; p=0.0746); neoadjuvant HR: 0.7 (95% CI:0.59–0.83; p= <0.0001)]. This difference in 5-year overall survival between TNBC patients receiving adjuvant and neoadjuvant treatment strategies was consistent across age groups, however changes in OS among diagnostic cohorts varied across age groups (**Figure 3**).

Overall survival also varied across age groups within molecular subtypes, with older patients having generally worse survival rates (**Supplementary Tables 3**–**6**). Survival rates tended to either improve or fluctuate, however, a declining trend was observed in the TNBC group among patients aged 60–69 years. In the youngest cohorts, 5-year survival rates exceeded 90% in HER2-/HR+ and HER2+/HR+ groups, while it reached 80% in the HER2+/HR- groups for the 2017-2018 diagnostic cohort. In contrast, 60-month overall survival of TNBC remained under 80% in patients aged under 50 years of age, with 74.15% during the 2017–2018 diagnostic period.

**Figure 4:**
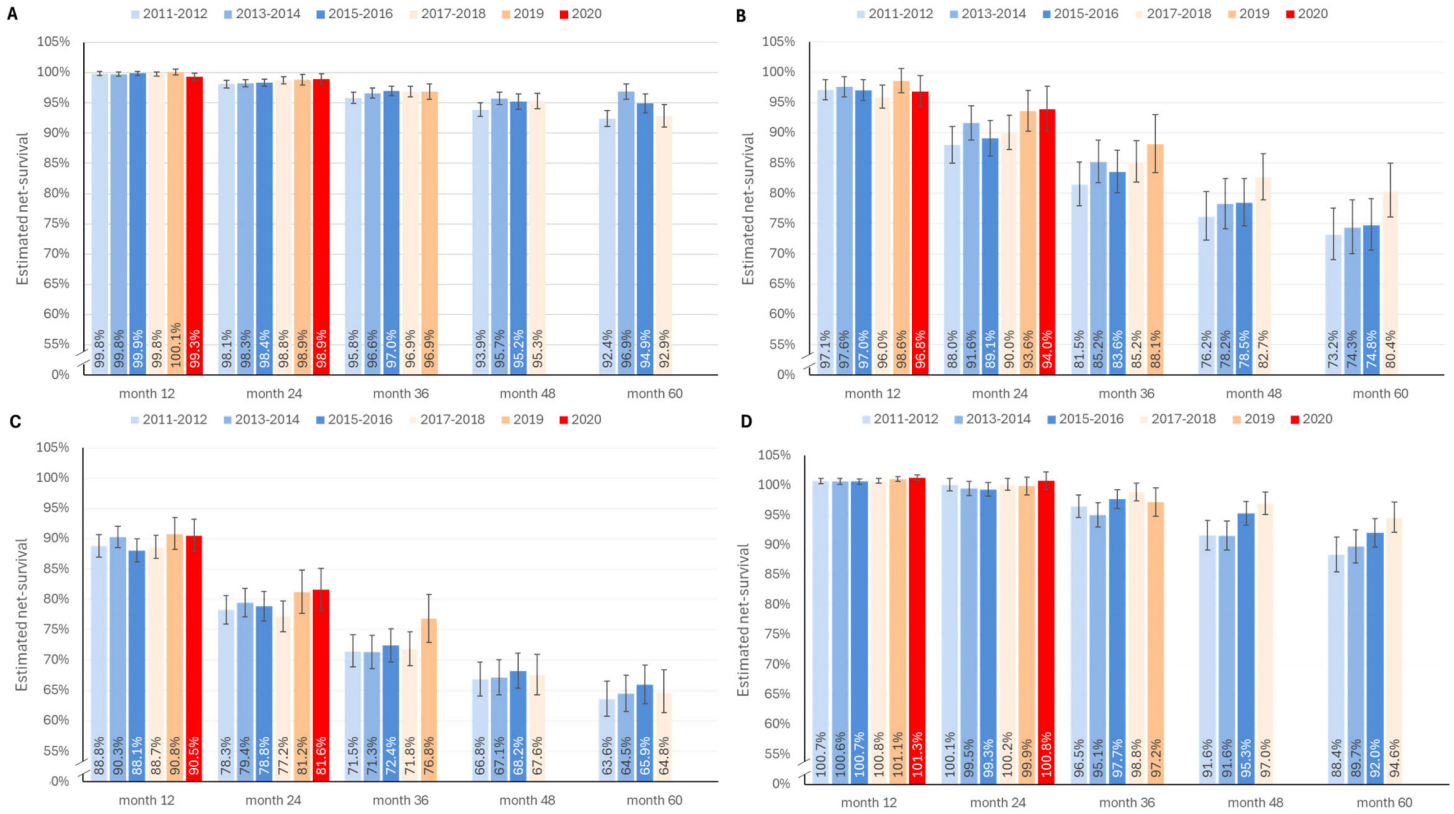
One- to 5-year net survival estimates for patients with HER2–/HR+ **(A)**, HER2+/HR– **(B)**, TNBC **(C)**, and HER2+/HR+ **(D)**, by different periods (from 2011–12 to 2020, survival rates for a given cohort is provided up to the longest follow-up time within the observation period). Error bars represent 95% confidence intervals. BC, breast cancer; HER2, human epidermal growth factor receptor 2; HR, hormone receptor; TNBC, triple-negative breast cancer.

Patients with HER2–/HR+ BC had the most favorable age-standardized net survival among all BC subtypes, with 1-year net survival exceeding 99% and 5-year survival exceeding 92% in all diagnostic periods (**Figure 4A**). Patients with HER2+/HR+ and HER2+/HR– BC had somewhat poorer net survival at 5 years, ranging from 88.3 to 94.6% and 73.2 to 80.4%, respectively (**Figures 4B, D**). Of note, net survival up to 3 years was higher in the HER2+/HR+ group, than in the HER2–/HR+ group. The lowest net survival was found in patients with TNBC whose 1-year survival was 88–90.5% and 5-year survival was as low as 63.6–65.8% (**Figure 4C**).

In general, net survival rates beyond 2 years tended to improve in patient cohorts diagnosed later during observation period, however, the differences were not statistically significant apart from the HER2+/HR+, where 4 and 5-year net survival improved in the 2017-2018 cohort compared to 2011-2012. Due to the limits of the observation period, we were not able to draw survival data for a full 5-year follow-up for patients diagnosed after 2017–2018. In the TNBC subgroup, there was an increase in 1- and 2-year net survival rates for the cohorts diagnosed in 2019 and 2020, however, these were not statistically significant. Three-year net survival also improved in the HER2+/HR– and TNBC cohorts in 2019, and 4- and 5- year net survival in the HER2+/HR– group in the 2017-2018 diagnostic period as compared to earlier diagnostic periods.

## Discussion

This nationwide, retrospective study was performed as part of the Hungarian HUN- CANCER EPI Multiple Cancer Epidemiology program to provide a deeper analysis into breast cancer subtypes than epidemiology results our team already published (16) and to examine breast cancer incidence and survival in Hungary between 2011 and 2020, focusing on major BC subtypes based on HR and HER2 status. The observation period included 2020 when the COVID-19 pandemic reached Europe.

As for the major BC clinical subtypes, the proportions of HER2+/HR–, HER2–/HR+, TNBC, and HER+/HR+ subtypes were 3.6%, 61.9%, 8.4%, and 6.2% of all BC cases in our study, respectively. Omitting cases with unknown subtypes, these proportions were 4.5%, 77.2%, 10.5% and 7.8%, respectively. Cancer registry data from the SEER program reported fairly similar proportions from the U.S. for the period 2016–2020: 4% for HER2+/HR−, 69% for HER2–/HR+, 10% for TNBC, and 10% for HR+/HER2+ (20). In the Italian AIRTUM network, 64.9% of patients had the full molecular profile for classification. Among these BC cases, 6.2% were HER2+/HR–, 66.2% were HER2–/HR+, 8.5% were TNBC, and 19.1% were HER+/HR+ (21). The EUSOMA international network of European breast centers also reported data on the distribution of BC subtypes among patients diagnosed between 2016–2021: 4.2%, 76.7%, 8.9%, and 10.1% of BC cases were as HER2+/HR–, HER2–/HR+, TNBC, and HER+/HR+ (22). Corresponding proportions reported by the Netherlands Cancer Registry were 5%, 74.9%, 10.9% and 9.3%, respectively (23). Therefore, we can state that our results are largely consistent with available international data, underscoring the validity of conducting additional survival analyses for these subtypes. Interestingly, one study from Slovakia analyzing pathology registry data reported a somewhat higher proportion for TNBC (12.2%) among patients with non-Roma ethnicity, and an even higher proportion among patients retrospectively identified as Roma (28.1%), although the latter was derived from a small sample of only 32 cases (24).

We observed a slight numerical increase in the proportion of HR+/HER2– and HR+/HER2+ subtypes and a decrease in TNBC subtype, and in the number of cases without identifiable chemotherapy received. Considering that we saw major changes in the proportion of TNBC patients receiving perioperative systemic treatment and that the classification was made indirectly based on the therapies administered, these changes in the proportion of molecular subtypes probably indicate developments in clinical practice and improving access to targeted therapies. For example, trastuzumab treatment already used as adjuvant therapy became reimbursed in Hungary in 2013 for the neoadjuvant setting, and the number of patients having received trastuzumab increased in the first half of our observation period (25).

In line with previous reports, TNBC and HER2+ tumors were more frequent among younger patients in our study, and HR+ BC became more and more dominant with increasing age (26, 27). In patients aged <40 years, about 50% had HR– malignancy and almost 25% had TNBC. Although the majority of incident BC cases are diagnosed in patients aged ≥50 years, the higher proportion of TNBC among younger women warrants a more thorough analysis of the behavior of this subtype across age groups, especially in light of the increasing incidence of BC observed among women younger than 50 years during the past decade in our cohort (16). In addition, TNBC is not a homogeneous entity, it can be subdivided into further molecular subtypes, and tumor-specific treatment options are still lacking in contrast to other major BC subtypes. Clinically, TNBC demonstrates an aggressive biological behavior with a greater proportion of histopathological grades 2 and 3 (28) and early distant recurrence rates are also more frequent, than with other types of BC (11). This was also reflected in our study: patients with TNBC had the poorest overall survival among all BC subtypes, with a 5-year OS rate of 61.4%.

The observed 5-year net survival rate for patients diagnosed between 2015–2019 was 87.8% in Hungary. The U.S. SEER program reported 90.8% relative survival for BC for the period 2013–2019 (20). The CONCORD-3 study published age-standardized 5-year BC net survival estimates for Europe for patients diagnosed between 2010–2014, ranging from 70.8% (Russia) to 89.1% (Iceland). Although age-standardization would limit direct comparison with our data, there were no estimates reported for Hungary from CONCORD-3. As a benchmark, rates in surrounding countries ranged from 74.8% (Romania) to 84.4% (Austria) (4). In addition to CONCORD-3 data, more recent net survival data were published by certain national cancer registries in Europe. Estimates from Slovenia showed a net survival rate of 87.6% for the diagnostic period 2012–2016 (29). According to the Polish National Cancer Registry, female BC relative survival was 78.8% in Poland in the 2010–2015 period (30). Net survival of BC was reported to be 87.5% in England in the diagnostic period of 2016–2020 (31) and 89% in the Netherlands for the 2015–2019 period (32). The German Centre for Cancer Registry reported relative survival rate of 88% for the years 2011–2018. Considering these survival rates, Hungarian BC net survival rates from our study are similar to those reported from Slovenia but do not reach the ones reported from Western Europe and US. There are some methodological caveats, though, when comparing data across these studies. First of all, there were significant differences in data collection methodology: most of the scientific literature reported population-based results relying on cancer registries, while our study was based on data collected by the Health Insurance Fund. Furthermore, net survival estimation methods may yield different outcomes (33), and even the estimates by the current gold standard Pohar Perme method may be impacted by the life tables data used for the given population (19).

Net survival rates of BC showed clear differences across molecular subtypes, with HR+/HER2– being associated with the highest 5-year survival rates (>90%) and TNBC with the lowest (61–65%). This is in line with the most recent report from SEER, although the U.S. survival rates were higher (94.8% for HR+/HER2– BC, 91% for HR+/HER2+ BC, 85.6% for HR-/HER2+ BC, and 77.6% for TNBC) (20). Five-year relative survival among Belgian women diagnosed in 2014 was reported to be 91.4%. The highest survival rates after 5 years were observed in the luminal A-like breast cancer group (96.8%), while TNBC was associated with a 5-year relative survival of only 77.4% (13). Similar to the U.S., the Belgian data also demonstrate better survival, than what we observed in Hungary, reaching a difference of more than 10% in the case of TNBC. Possible explanations of such divergent outcomes could include dissimilarity in distribution of cancer stages at discovery or differences in healthcare and therapies applied, however more detailed data should be gathered to study these factors further.

While net survival helps interpret and compare survival probabilities associated with BC, overall survival rates are helpful and intuitive measures for clinical practitioners and patients. The observed overall survival for patients diagnosed between 2015 and 2019 was 74.2% for the whole BC patient population, however, it varied across subtypes. The HER2+/HR+ group had the highest 5-year survival rates (86.5%), followed by HER2–/HR+ (79.1%), HER2+/HR– (71.9%), and TNBC (61.4%). For comparison, 5-year overall BC survival in U.S. was estimated to be between 81.7% and 83.5% between 2010 and 2014 (34). A study analyzing BC patients’ data from the American College of Surgeon and American Cancer Society’s National Cancer Database (NCDB) between 2010–2014 reported similar order of different molecular subtypes in terms of 5-year overall survival, albeit with somewhat higher rates even for TNBC (exceeding 70% in contrast to the observed 61.4% in our study) (35). In another U.S. study, TNBC was associated with worse relative and overall survival, than HR+/HER2− tumors, even after adjusting for age, stage, race, use of adjuvant chemotherapy, tumor size, grade, and nodal status (36). The study also described an increased risk of death within 2 years of diagnosis that diminished in the later years of follow-up period, which is consistent with TNBC OS decreasing to 76.37% by year 2 in our study. Besides the generally worse survival prospects of TNBC patients, the fact that 5-year survival rates did not even reach 75% in patients aged <50 years is particularly disappointing given that they represent the most active population and TNBC is more common among them, than in later age groups. The need to improve outcomes in TNBC is also emphasized by the observed decrease in survival rates over time in the age group of 60–69 years. Survival rates may differ depending on the stage of cancer at diagnosis. Depending on tumor stage, a Canadian study reported 5-year survival rates between 96.5% (stage I) to 36.6% (stage IV) for HER2+, 94.7% to 24% for HR+, and 93.3% to 7.4% for TNBC (26). Data on cancer stage were not available in the NHIF database for direct comparisons in stage-related survival rates, however, we observed diverging survival trajectories in TNBC patients based on the setting of systemic therapy initiation (adjuvant or neoadjuvant). Patients who had surgery before receiving systemic treatment had better survival (82.4%), than those receiving neoadjuvant treatment (67.5%). According to current ESMO guidelines, first-line surgery is recommended only for small T1a and T1b tumors without nodal involvement, in other cases, therapy should start with neoadjuvant treatment (14). Although we did not observe cancer stages directly and patients with advanced stage TNBC may have been included in the neoadjuvant group, recently published data from the Swedish Cancer Register from the period of 2008 to 2020 showed that 10.9% of non-metastatic TNBC patients undergoing neoadjuvant treatment were diagnosed in stage I as opposed to a 43.5% proportion seen among patients receiving adjuvant treatment (37). While typical diagnostic stages may vary with time and region, these data point out that our cohort of patients receiving neoadjuvant therapy may have also included some stage I (T1c) cases which makes the 67.5% overall survival rate among patients starting their systemic therapy in the neoadjuvant setting even more concerning.

Besides difference in the ratios of clinical stages at the time of breast cancer diagnosis, several factors and their complex interactions should be considered that may underly regional dissimilarities in prognosis. Population age structure may be quite different between countries, which may impact population level outcomes as poorer prognosis has been observed in breast cancer patients over 75 years of age. This phenomenon may be attributed to multiple reasons including delayed diagnosis, worse general physical condition and presence of more chronic comorbidities, as well as polypharmacy and drug side effects that can drive treatment nonadherence (38). Nevertheless, net survival analyses may account for differences in age distribution by using an age-standardized approach. Other demographic factors such as race may also contribute to differences in survival. For example, TNBC has been described to have worse outcome in African American patients, however the underlying reasons have not been full untangled due to the interplay of biologic and environmental factors such as genetic susceptibility, co-morbid conditions, exposure to environmental risks, socio-economic status and access to healthcare (39, 40). Prevalence of risk factors related to breast cancer survival rate, such as obesity and smoking may also vary from country to country, as well as other lifestyle-related factors including rate of physical activity, age at first pregnancy, breastfeeding rate and duration, or the use of hormonal medicinal products (41, 42). Of note, socio-economic disparities and access to medical care have been identified as important aspects in underlying unequal clinical outcomes not only among different countries, but also within the borders of highly developed states (43–45). A recent study reported that patients from the most deprived areas were 10% less likely to receive HER2-targeting treatment than those from the least deprived territories in the UK even though access to the treatment is freely available for all patients (46). This example illustrates that significant inequalities of treatment access may be present even when reimbursement is equally ensured, although financing of therapies may also largely vary among countries. In the case of 4 Central Eastern-European countries, there were major disparities in the status of reimbursement of novel oncological pharmacotherapies in 2022: while Czechia covered 64% of the studied indications, the rate was 51% in Poland, 40% in Hungary, and only 19% in Slovakia despite the largely similar economical situation of the countries (47). Handling all these factors require comprehensive, data informed strategy development from health policy makers to effectively integrate health promotion and education, preventive public health initiatives, and broad access to streamlined cancer diagnostics, medical care, follow-up and modern therapies.

Our study included data from the year when the COVID-19 outbreak reached Europe and countries introduced various measures to control viral spread and avoid the critical overburdening of health care institutions. During the public health emergency, the usual access to medical care was disrupted across Europe, including screening programs (48). Such a change in healthcare access may have had an impact on the detection time and stage of breast cancer and may have affected patient prognosis by increased mortality due to the newly emerging COVID-19 infections or the disturbance in the follow-up of chronic conditions. In our case, the pandemic related public health measures affecting the provision of healthcare probably contributed to the lower number of incident cases identified in the 2020 patient cohort, otherwise the number of new cases was approximately stable across the period. Several risk factors for COVID-19 mortality overlap with those for breast cancer, including older age, obesity, and certain comorbidities. As a result, the excess mortality observed during the pandemic may have disproportionately affected individuals who were at risk of developing breast cancer, potentially leading to a lower recorded incidence in 2020–2021. This could be explained by the fact that some cases were never diagnosed due to premature COVID-19-related deaths occurring before cancer detection. Besides, despite the challenging year of 2020 and decreased breast cancer screening and surgical care rates reported from this period from Hungary, we did not observe significant change in short term overall and net survival of BC patients at any subtype (49).

The main strength of our study lies in the comprehensive nature of the NHIF database which made it possible to estimate survival rates and the relative frequency of BC subtypes indirectly on a nationwide level, ensuring the generalizability of the results. As the NHIF database primarily captures healthcare events based on ICD-10 codes, therapies applied, and payor-defined intervention codes in the focus setting of the study (i.e. ambulatory and hospital-based oncology-related healthcare), no efforts were made to directly assess tumor stage or subtype. Instead, we applied an indirect approach to identify HR and HER status based on administered therapies. The validity of this approach is supported by the fact that the resulting BC subtype proportions were largely consistent with international data. We were not able to classify the whole BC population into subtypes based on the received therapies received only, which may have resulted in an underestimation of certain subtypes such as very early-stage TNBC which may be treated with surgery and no systemic treatment. Very early death after diagnosis or clinical trial participation may have also led to missing subtype-relevant treatment. Of note, missing data in cancer registries may not be completely at random either, therefore, the frequent practice of analyzing only complete records may overestimate overall survival as the lack of certain clinical data may be a result of mortality before the completion of all diagnostic procedures (34). Considering that our survival analysis covered the whole BC patient population and case definition already considered death close to tumor discovery, our results may provide a more reliable estimate, than certain registry-based reports. Still, we cannot exclude that a similar phenomenon affected our subtype-specific survival rates. Importantly, even if net survival rates carry similar inaccuracies as previously reported data, this would not hinder the interpretation of results pertaining to trends in survival rates, and it should be noted that accuracy of survival estimates for all subgroups is further improved by the completeness of time of death data for deceased patients in our study. Given the descriptive nature of our analysis focusing on a single country and limited period of time, no external age standardization was applied on net survival which should be kept in mind when considering international data and changes over time.

## Conclusion

Our nationwide study examined the most recent developments in BC survival and potential differences across BC subtypes in Hungary using a comprehensive health insurance fund database. The proportion and age distribution of molecular subtypes according to HR and HER2 status were in line with previous reports, with a lower proportion of HR+ tumors in younger patients which confirms our patient identification approach. Overall, survival prospects for BC patients improved during the study period approaching recent survival rates in certain Western European countries, however, they did not reach those seen in Nordic and North American countries. Despite improvements across all molecular subtypes, there is still a significant need for increasing BC survival rates, especially for more aggressive tumor types including HER2+/HR– and TNBC which account for a higher proportion of BC cases in younger ages.

## Data Availability

The original contributions presented in the study are included in the article/**Supplementary Material**. Further inquiries can be directed to the corresponding author.
